# Periprocedural Outcomes of Rotational Atherectomy-Assisted Balloon Angioplasty in Isolated Atherosclerotic Popliteal Artery Lesions: The ISO-POP Trial

**DOI:** 10.3390/jcm12082797

**Published:** 2023-04-10

**Authors:** Konstantinos P. Donas, Anastasios Psyllas, Apostolos G. Pitoulias, Majid Kazemtash, Firouza Dahi, Nizar Abu Bakr, Grigorios Korosoglou

**Affiliations:** 1 Rhein Main Vascular Center, Department of Vascular and Endovascular Surgery, Asklepios Clinic Langen, Divisions of Vascular Surgery Asklepios Clinics Seligenstadt and Wiesbaden, 63225 Langen, Germany; 2 Department of Vascular and Endovascular Surgery, Marienhospital Wesel, 46483 Wesel, Germany; 3 Cardiology, Vascular Medicine and Pneumology, GRN Hospital Weinheim, 69469 Weinheim, Germany

**Keywords:** isolated popliteal artery, rotational atherectomy, endovascular treatment, claudication, critical limb ischemia

## Abstract

Background: Treatment of calcified popliteal artery lesions represents an ongoing challenge for vascular specialists. Biomechanical forces of external compression, torsion and elongation that occur with locomotion in the popliteal segment can lead to stent fractures and occlusions. The aim of our study was to assess the procedural success rate of atherectomy in combination with balloon angioplasty for isolated calcified popliteal artery lesions. Methods: Between January 2020 and December 2022, 62 patients with isolated atherosclerotic lesions of the popliteal artery underwent endovascular treatment by use of rotational atherectomy (Phoenix, Philips USA, (subgroup A) or Jetstream, Boston USA, (subgroup B), atherectomy systems) and additional balloon angioplasty in two vascular centers. The primary outcome measures were: 1. periprocedural clinical and technical success (<30% residual stenosis and no need for bailout stenting due to flow-limiting dissection) and 2. postprocedural increase in the ankle brachial index of more than 0.1. Results: The overall rate of bailout stenting was 4.8%, whereas the procedural success rate was 98.4%. The rate of procedural complications included 3.7% and 5.7% peripheral embolizations in the subgroups A and B, respectively, and no vessel perforations were noted. All embolizations were successfully treated by catheter aspiration or capture in the pre-treatment placed filter system. In addition, 1 (3.7%) pseudoaneurysm in the groin was reported in subgroup A and treated by surgical means. Median ABI of the affected limbs improved from 0.55 (0.2) to 0.70 (0.2) in subgroup A and from 0.50 (0.2) to 0.95 (0.1) in subgroup B (DABI of 0.15 versus 0.45, *p* < 0.001). Conclusions: The combination of rotational atherectomy and balloon angioplasty in the popliteal artery showed reproducible outcomes in 2 centers, with low incidence of complications and low rates of bail-out stenting. These findings may contribute to more liberal use of such devices especially in segments with high risk for stent factures and occlusions.

## 1. Introduction

Despite recent advances of endovascular therapies, the treatment of calcified popliteal artery lesions represents an ongoing challenge for vascular specialists. Drug coated balloons (DCB), while effective in soft atherosclerotic lesions, are mechanically challenged in calcified lesions to expand completely. In addition, the calcium represents a barrier to drug uptake and a challenge to stents resulting in stent malposition, compression, which may occur even with dedicated interwoven stents [1]. The main reasons for these findings are biomechanical forces of external compression, torsion and elongation that occur with locomotion in the popliteal segment. Minimally invasive atherectomy aims to enhance the effectiveness of endovascular therapy by providing luminal gain without barotrauma, since the latter can cause dissection, recoil and disruption of the elastic lamina, leading to restenosis [2]. Thus, atherectomy enables more homogeneous balloon expansion during low pressure angioplasty, facilitating higher deliverability of drugs into the vessel wall during subsequent drug-coated-balloon (DCB) angioplasty [3,4,5]. Due to the plethora of types of atherectomy and the mixed published populations, including lesions from the common femoral artery up to below-the-knee (BTK) vessels, however, definitive conclusions cannot be safely drawn about the incremental values of this vessel preparation tool in atherosclerotic lesions for specific vessel segments. Specifically, for isolated popliteal lesions the data are scant. Thus, limited evidence exists in the field of the popliteal artery and refers to directional and orbital atherectomy types, only [6].

The aim of our study was therefore to assess the procedural success rate of rotational atherectomy in combination with balloon angioplasty for isolated calcified popliteal artery lesions.

## 2. Methods

Study Design: We performed a dual center, prospective study, aiming to assess procedural data in patients treated using rotational atherectomy devices for the treatment of isolated popliteal artery lesions. Our trial was registered on the German Clinical Trials Register website (DRKS00016708). Approval was obtained from our local ethics committee (S-100/2017) of the University of Heidelberg and all patients provided written informed consent.

Patient Population and Endovascular Procedures: Demographic data and cardiovascular risk factors were analyzed.

All procedures were performed or supervised by experienced interventional angiologist (G.K.) or endovascular surgeons (K.P.D., N.A.B.) board certified for the endovascular treatment of PAD by the German Societies of Angiology and Cardiology or Vascular Surgery. Details regarding our endovascular protocols were reported previously [7]. In short, lesions were passed with an antegrade or, when failed, with a retrograded approach [8]. Generally, care was taken to maintain the wire in the intraluminal space throughout the lesion. In this regard, the guidewires with high penetrating power loaded on straight and angled support catheters were used if necessary to facilitate intraluminal passage at the discretion of the operator. In case of a failed antegrade passage because the wire entered or remained in the subintimal space, the retrograde approach was liberally chosen in the decision of the physician after puncture of the pedal or crural vessels, to maintain intraluminal passage from retrograde [9]. If intraluminal passage could not be obtained from either antegrade or retrograde, atherectomy was deferred in accordance with the instructions for use (IFU), which warn against usage of such devices in the subintimal space.

The Jetstream atherectomy (Boston Scientific, Marlborough, MA, USA) device consists of a single use atherectomy catheter with a control pod and a reusable compact power console. The single use catheter consists of a five-flute, front-end cutting tip, which rotates at 70,000–73,000 RPM. Two expandable catheter cutters XC 2.4/3.4 and XC 2.1/3.0 were used for lesion debulking, inserted over a 0.014” guide wire. The system combines dynamic and continuous aspiration of fragmented debris to reduce the risk of distal embolization.

The Phoenix rotational atherectomy system (Philips, San Diego, CA, USA) consists of the catheter, which houses the cutter at its distal tip. The cutter rotates at high speed between 10,000 and 12,000 rpm, so that fragmented debris is removed due to strong suction forces. This obviates the need for filter protection. The Phoenix system is delivered over a 0.014-inch guide wire and can be used for the treatment of vessels with a diameter between 2.4 mm and 7 mm. In our study, typically the 2.4 mm(7F) deflecting device (appropriate for arteries with diameters > 4.5 mm) and the 2.2 mm(6F) tracking or deflecting devices (appropriate for arteries with diameters of 3.0–6.0 mm) were used in the popliteal artery.

Patients with whom the Jetstream atherectomy was used are referred to as ‘Subgroup A’, whereas patients who underwent Phoenix atherectomy are referred to as ‘Subgroup B’. The Phoenix or the Jetstream atherectomy devices were used prior to balloon angioplasty.

Following atherectomy, lesions were typically treated using low-pressure plain old balloon angioplasty (POBA). In most cases, POBA was followed by drug coated balloon (DCB) angioplasty. In case of persistent recoil or flow limiting dissections, self-expanding bare metal stents were implanted.

Duplex sonography was performed on the day after the procedure to investigate vessel patency and to detect potential complications. Furthermore, an Ankle-Brachial Index (ABI) measurement was performed before and at the day after the procedure.

Between January 2020 and December 2022, 62 patients with isolated atherosclerotic lesions of the popliteal artery underwent endovascular treatment by use of rotational atherectomy (Phoenix or Jetstream atherectomy systems) and additional balloon angioplasty in 2 vascular centers. Inclusion criteria of the study were symptomatic PAD with lifestyle limiting claudication or critical limb threatening ischemia (CLTI) due to (i) isolated involvement of the popliteal artery from the first P1 up to the third segment P3 of the popliteal artery, (ii) occlusion or high-grade stenosis of more 70% based on computed tomography angiography (CTA) or angiography, (iii) atherosclerotic lesions with at least mild calcifications by fluoroscopic or CT-angiographic criteria. Patients with other relevant ipsilateral lesions requiring treatment and acute or subacute thrombotic occlusions were excluded. The primary outcome measures were (1) periprocedural clinical and technical success (<30% residual stenosis and no need for bailout stenting due to flow-limiting dissection) and (2) postprocedural increase in the ankle brachial index of more than 0.1.

Analysis of Angiographic Data: The TASC classification was used for determining the lesion complexity, localization and calcification [10]. The degree of calcification was categorized by visual criteria in mild, moderate or severe.

Statistical Analysis: Analysis was performed using commercially available software IBM SPSS Statistics, 28.0. (IBM Corp., Armonk, NY, USA). Nominal and categorical variables are presented as counts and proportions and were analyzed by Pearson’s chi square test, as appropriate. Following the Shapiro–Wilk normality test analysis all continuous variables were found to have skewed distribution (*p* < 0.05). Further analysis of continuous variables was made by non-parametric Mann–Whitney U-test and all data in text and tables are presented as median and interquartile range (median-IQR). All tests were two-tailed and significant level of difference was set at *p* < 0.05.

## 3. Results

Demographic Characteristics: Table 1 summarizes demographic, angiographic, and procedural data in 27 and 35 patients and in subgroups A and B, respectively, who underwent rotational atherectomy of atherosclerotic isolated popliteal arteries. Subgroup A was treated by endovascular surgeons and subgroup B by angiologists.

Patients were 77(14) years old, and 35(56.5%) were male. Fifteen percent of the patients had diabetes and 22(35.5%) had history of CAD. Mean GFR was 56(20)mL/min/1.73 m^2^, 9(15%) patients were on chronic hemodialysis and 54(87.1%) were on a lipid lowering therapy. Most clinical parameters were similarly distributed in subgroups A and B, except for patients who smoked, who were more frequently represented in subgroup A, and patients with dyslipidemia, more frequently represented in subgroup B.

Clinical Presentation and baseline ABI values: Of 62 patients, 28(45.2%) had lifestyle limiting claudication Rutherford category III (RC-III), 11(17.7%) had ischemic rest pain (RC-IV) and 23(37.1%) had ischemic ulcerations (RC-V). No differences were observed between the 2 subgroups.

Lesion Characteristics and Distal Run-off: In all cases the target vessel was the popliteal artery. Involvement of more than one segments of the popliteal artery was present in 34(54.8%) of the cases, including 17(27.4%) with involvement of the P1 and P2, 1(1.6%) with involvement of the P2 and P3 and 16(25.8%) with involvement of all P1, P2 and P3 segments.

Lesion length was 72.5 (39.0, IQR: 55.0–90.0) mm and occlusions were present in overall 42(67.7%) of the patients.

Lesion calcification was mild in 4(6.5%), moderate in 17(27.4%) and severe in 41(66.1%) of the patients. In addition, the number of distal patent run-off vessels was 3 in 7(11.3%) patients, 2 in 41(66.1%) and an only single vessel run-off was present in 14(22.6%) of the patients.

Lesion length, calcification and the number of distal run-off vessels were similar between subgroups A and B (*p* = NS). Involvement of more than one popliteal subsegments on the other hand, was more frequent in subgroup B (68.6% versus 37.0%, *p* = 0.03), whereas total occlusive lesions were more frequently found in subgroup A (88.9% versus 51.4%, *p* = 0.005).

Technical Details, Adjunctive Treatments and Complications: Primary atherectomy as vessel preparation was performed in all cases by use of the Jetstream XC 2.4/3.4 mm and XC 2.1/3.0 mm catheter systems of the subgroup A, whereas the 2.4 mm deflecting, 2.2 mm deflecting and 2.2 mm tracking Phoenix atherectomy devices were used in the subgroup B, in 10(28.6%), 11(31.4%) and 14(40.0%) cases, respectively.

Distal embolic protection devices were used by decision of the physician in case of poor run off (only 1 patent BTK vessel to the foot) and this was preferred in 10(37%) of the patients in subgroup A and in no cases in subgroup B (*p* < 0.001). In three patients the Emboshield NAV6 (Abbott, CA, USA) was used and in the other 7 the Spider FX (Medtronic, Santa Rosa, USA) in subgroup A.

Adjunctive treatment after debulking, was performed using DCB angioplasty in 23(85.2%) and in 35(100%) cases of the subgroups A and B (*p* = 0.02), whereas bail-out stenting was performed in 1(3.7%) and 2(5.7%) cases, respectively (*p* = NS). Procedural success was achieved in 26(96.3%) and 35(100%) of the patients, respectively (*p* = NS).

The rate of procedural complications included 1(3.7%) and 2(5.7%) peripheral embolizations in the subgroups A and B, respectively, and no vessel perforations. All embolizations were successfully treated by catheter aspiration and remained asymptomatic. In addition, 1(3.7%) pseudoaneurysm in the groin was reported in subgroup A and treated by surgical means.

Primary endpoint and ABI measures: The overall rate of bailout stenting was 4.8%, whereas the periprocedural success rate was 98.4%.

Median ABI of the affected limbs improved from 0.55 (0.2) to 0.70 (0.2) in subgroup A and from 0.50 (0.2) to 0.95 (0.1) in subgroup B. ABI increases were significant in both subgroups, but the median ABI difference was higher in subgroup B (DABI of 0.15 versus 0.45, *p* < 0.001).

Major adverse events: Two of 27 patients of subgroup A experienced major adverse events (MAEs) during hospital stay. One patient had pneumonia and 1 underwent coronary angiography due to non-ST elevation myocardial infraction. In subgroup B, no MAEs were reported during hospital stay. The overall mortality rate was 0%.

**Figure 1:**
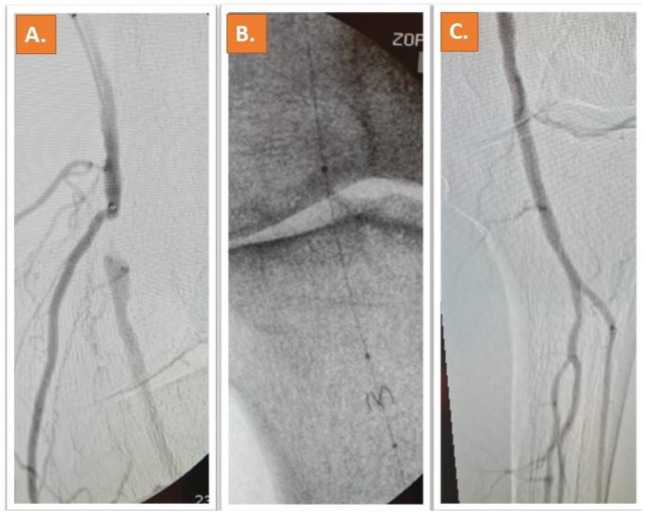
A chronic moderately calcified occlusion of the popliteal artery can be depicted in (**A**). Treatment was successfully performed with Jetstream rotational atherectomy after placement of a filter system (**B**). The result after Jetstream-assisted atherectomy and DCB can be appreciated in (**C**) [11].

**Figure 2:**
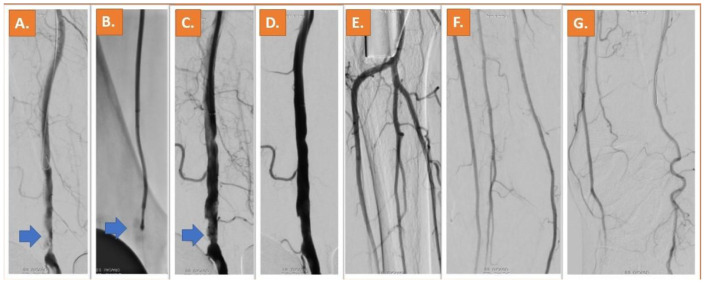
A calcified lesion in the P1 segment of the popliteal artery can be depicted in (**A**) (blue arrow), in a female 81 years old patient with lifestyle limiting claudication (RC4). After 2.4 mm deflecting Phoenix atherectomy (**B**), significant lumen gain is obtained (**C**). The result after atherectomy and DCB including the perfusion of the peripheral vessels can be appreciated in (**D**–**G**) [11].

Representative Cases: Two cases of patients who underwent Jetstream and Phoenix atherectomy are shown in Figure 1 and Figure 2.

## 4. Discussion

The present 2-center study demonstrates, to our knowledge for first time in the current literature, safe and reproducible periprocedural outcomes for patients who undergo rotational atherectomy in isolated popliteal lesions. The need for bailout stenting due to recoil or flow-limiting dissection is low 4.8%, considering that overall, more than 50% of the treated cases were calcified CTOs. Regarding complications, no iatrogenic rupture or pseudoaneurysm formation in the popliteal area was observed in both subgroups. In addition, the rate of peripheral embolization was less than 5%.

Previous studies, using directional atherectomy for the treatment of isolated popliteal lesions reported on vessel injury and distal embolization in 5% of the cases [12]. Considering these data, the use of rotational atherectomy in the popliteal artery appears relatively safe in terms of vessel injury, whereas embolization rates are comparable. In another study, using directional atherectomy for the treatment of isolated popliteal arteries, the procedural success rate was slightly lower (84.4%), but the rate of bail-out stenting was similar (3.7%), as in our study [13]. However, this trial included ~70% patients with lifestyle limiting claudication and possibly less complex and calcified lesions based on the reported lesion characteristics (5.8 versus 7.3 cm lesion length, 23.5% versus 66% total occlusive lesions). In a recent multi-center collaborative by Troisi et al., in 409 patients with symptomatic PAD due to isolated popliteal artery lesions or occlusions, DCB alone or combined with atherectomy and if required stent placement was found to be superior to POBA in terms of primary patency [14]. However, only 17(4.1%) patients of this cohort underwent rotational atherectomy, making it difficult to withdraw safe conclusions on the value of this modality in isolated popliteal artery lesions.

To our knowledge the present study is the first prospectively reporting on procedural outcomes with 2 different rotational atherectomy devices for the treatment of isolated popliteal lesions. As with previous reports, which used orbital atherectomy [15], our study showed that rotational atherectomy is safe and effective for the treatment of such patients.

All atherectomy devices probably carry different risks for distal embolization, which depend both on device properties and the composition of the atherosclerotic lesions. Therefore, distal filter protection devices need to be considered, depending on the type of the atherectomy device and the anticipated lesion composition. The Emboshield NAV6 (Abbott Vascular, CA, USA) is usually recommended for rotational atherectomy devices such as the Jetstream, whereas the SpiderFX (Medtronic, Santa Rosa, CA, USA) is rather recommended with directional devices. Vasodilators may also be considered with such procedures to counteract vasopasm, triggered during plaque modification [16]. Krishnan et al., 2021 [17] compared the safety and efficacy between the SpiderFX EPD and Emboshield NAV6 filter in the collection of embolic debris created from lower limb atherectomy procedures in patients with PAD, finding no significant clinical differences between the 2 embolic protection devices in terms of clinical outcomes. In our study, either the Emboshield NAV6 or the SpiderFX devices were used in subgroup A. Notably, ex-vivo preparation of the Emboshield NAV6 prior to its insertion in the vessel may be more time consuming compared to the SpiderFX device, however the prior may be more stable distal to the lesion after deployment.

Increased treatment length is associated with an increased risk of atherectomy-associated complications. This was the finding of a retrospective review analyzing 729 atherectomy procedures performed between January 2011 and December 2015 in the Southern California Vascular Outcomes Improvement Collaborative [18]. Probably the short length of the treated popliteal artery of about 7 cm may have influenced the low rate of complications in our study, which may be higher in case of longer and more complex lesions or with in-stent restenosis or occlusion [19].

In addition, a recent meta-analysis highlighted to the superiority of drug eluting stents (DES) for the treatment of femoropopliteal segments, compared to DCB, pointing to the need for more thorough lesion preparation, for example, using atherectomy in combination with DCB in order to obtain as good results as with DES [11]. Since the popliteal artery is considered as a no-stent zone due to above mentioned anatomical considerations, this important meta-analysis re-enforces the usefulness of our combined atherectomy-assisted angioplasty approach for the treatment of isolated popliteal artery lesions.

Finally, another new finding of our study is that the discipline of the physician did not influence peri-procedural success and outcomes. All patients of subgroup A were treated by vascular surgeons, whereas patients of subgroup B were treated by angiologists. Despite slight differences in terms of lesion characteristics, outcomes were comparable and complication rates were low in both subgroups.

### Limitations

Our study has some limitations. First, no control arm was present of lesions treated without atherectomy. This arm would have been necessary to assess the incremental value of atherectomy compared to angioplasty alone. Such comparative studies, investigating the impact of atherectomy in such lesions, merit further investigation in future trials. In addition, follow-up data are currently not available, which is a limitation. However, the goal of the present study was to analyze procedural outcomes, whereas long-term outcomes may largely depend both on the use of debulking but also on the other tools used for the endovascular treatment of the lesions, such as the additional use of specialty balloons and DCB angioplasty. In addition, the presented outcomes are related to 2 specific rotational systems and can probably not be extrapolated to other rotational atherectomy devices. Finally, even if the present study includes the largest series in the literature to our knowledge with use of atherectomy in popliteal arteries in 62 patients, the sample size is relatively low to allow for safe comparisons between subgroups and future studies are warranted in this context.

In conclusion, the role of atherectomy devices for plaque modification and debulking as an adjunct to standard endovascular treatment options has been increasingly adopted particularly for complex and calcified lesions by vascular and endovascular specialists. Especially in the popliteal artery, which is considered as a no-stent zone due to biomechanical concerns and its potential use as a bypass graft landing zone, the use of atherectomy may aid reducing barotrauma and dissection, thus reducing the rates of bail-out stenting. In our current two-center study, we could demonstrate that the rate of complications as well as the rate of stent placement are equally low when using 2 different rotational atherectomy devices. This may add a milestone for the more liberal use of such devices especially in ‘no-stent’ segments.

## Figures and Tables

**Table 1:** Demographic and procedural data in patients with isolated popliteal artery lesions.

	Total *n* = 62	Subgroup A *n* = 27	Subgroup B *n* = 35	Subgroups A vs. B *p*-Values
Age (median years, IQR)	77–14	75–15	79–8	0.582
Age above median (*n*, %)	30–48.4	16–59.3	14–40.0	0.2
Male gender (*n*, %)	35–56.5	15–55.6	20–57.1	0.901
Smoking (*n*, %)	40–64.5	24–88.9	16–45.7	<0.001
Hypertension (*n*, %)	61–98.4	27–100	34–97.1	0.376
Dyslipidemia (*n*, %)	47–72.3	16–59.3	31–88.6	0.008
Diabetes (*n*, %)	14–14.8	4–22.6	10–28.6	0.199
Coronary artery disease (*n*, %)	22–35.5	7–25.9	15–42.9	0.167
GFR (median mL/min, IQR)	56–20	65–12	55–25	0.231
Moderate Chronic renal failure (*n*, %)	29–48.3	7–25.9	22–66.7	0.002
Dialysis (*n*, %)	9–15.0	6–22.2	3–9.1	0.156
Statin (*n*, %)	54–87.1	24–88.9	30–85.7	0.712
ACE inhibitor (*n*, %)	27–43.5	6–22.2	21–60.0	0.003
Antiplatelet or DOAC (*n*, %)	61–98.4	27–100.0	34–97.1	0.238
ABI preop (median, IQR)	0.5–0.2	0.55–0.2	0.5–0.2	0.434
Lesion length (median mm, IQR)	72.5–39	80–15	65–45	0.182
Lesion length above median (*n*, %)	31–50.0	15–55.6	16–45.7	0.442
Occlusion (*n*, %)	41–66.1	24–85.2	18–51.4	0.005
Target popliteal artery segment (*n*, %)				
P1 alone	14–22.6	9–33.3	5–14.3	0.030
P2 alone	11–17.7	8–29.6	3–8.6	
P3 alone	3–4.8	0–0.0	3–8.6	
P1 + 2	17–27.4	4–14.8	13–37.1	
P2 + 3	1–1.6	0–0.0	1–2.9	
P1 + 2 + 3	16–25.8	6–22.2	10–28.6	
Involvement of P2 segment (*n*, %)	45–72.6	18–66.7	27–77.1	0.359
Run-off crural vessels (*n*, %)				
1	14–22.6	9–33.3	5–14.3	0.084
2	41–66.1	17–63.0	24–68.6	
3	7–11.3	1–3.7	6–17.1	
Visual calcification score (*n*, %) (none/mild/moderate/severe 0–3)				
Class 0 & 1	21–33.9	6–22.2	15–42.9	0.089
Class 2 & 3	41–66.1	21–77.8	20–57.1	
Use of DCB (*n*, %)	58–93.5	23–85.2	35–100.0	0.019
POBA (*n*, %)	27–43.5	27–100.0	0–0.0	<0.001
Use of stent (*n*, %)	3–4.8	1–3.7	2–5.7	0.715
Use of filter (*n*, %)	10–16.1	10–37.0	0–0.0	<0.001
Aspiration thromboembolectomy (*n*, %)	3–4.8	1–3.7	2–5.7	0.715
Final technical success (residual stenosis <30%)	61–98.4	26–96.3	35–100.0	0.251
Improvement of ABI (*n*, %)	61–98.4	26–96.3	35–100.0	0.251
Perioperative complications (*n*, %)				
Embolization	3–4.8	1–3.7	2–5.7	0.490
Access	1–1.6	1–3.7	0–0.0	
ABI postop (median, IQR)	0.85–0.3	0.70–0.2	0.95–0.1	<0.001
ABI postop—preop difference (median, IQR)	0.31–0.3	0.15–0.1	0.46–0.2	<0.0001
Primary endpoint * (median, IQR)	54–87.1	23–85.2	31–88.6	0.693
Secondary endpoint ^#^ (median, IQR)	61–98.4	26–96.3	35–100.0	0.251

* patency of target lesion with atherectomy plus POBA or DCB and absence of bail-out stenting. ^#^ patency of target lesion with bail-out stenting.

## Data Availability

The data presented in this study are available on request from the corresponding author.
